# A Model for Studying the Hemostatic Consumption or Destruction of Platelets

**DOI:** 10.1371/journal.pone.0057783

**Published:** 2013-03-07

**Authors:** Mark R. Dowling, Emma C. Josefsson, Katya J. Henley, Benjamin T. Kile, Philip D. Hodgkin

**Affiliations:** 1 Immunology Division, The Walter and Eliza Hall Institute of Medical Research, Parkville, Victoria, Australia; 2 Cancer and Hematology Division, The Walter and Eliza Hall Institute of Medical Research, Parkville, Victoria, Australia; 3 Department of Medical Biology, The University of Melbourne, Parkville, Victoria, Australia; Albert Einstein College of Medicine, United States of America

## Abstract

A fundamental issue in understanding homeostasis of the hematopoietic system is to what extent intrinsic and extrinsic factors regulate cell fate. We recently revisited this issue for the case of blood platelets and concluded that platelet life span is largely regulated by internal factors, in contrast to the long-held view that accumulated damage from the environment triggers clearance. However, it is known that in humans there is an ongoing fixed requirement for platelets to maintain hemostasis and prevent bleeding; hence a proportion of platelets may be consumed in such processes before the end of their natural life span. Whether it is possible to detect this random loss of platelets in normal individuals at steady-state is unknown. To address this question, we have developed a mathematical model that independently incorporates age-independent random loss and age-dependent natural senescent clearance. By fitting to population survival curves, we illustrate the application of the model in quantifying the fixed requirement for platelets to maintain hemostasis in mice, and discuss the relationship with previous work in humans. Our results suggest a higher requirement for platelets in mice than in humans, however experimental uncertainty in the data limits our ability to constrain this quantity. We then explored the relationship between experimental uncertainty and parameter constraint using simulated data. We conclude that in order to provide useful constraint on the random loss fraction the standard error in the mean of the data must be reduced substantially, either through improving experimental uncertainty or increasing the number of experimental replicates to impractical levels. Finally we find that parameter constraint is improved at higher values of the random loss fraction; thus the model find utility in situations where the random loss fraction is expected to be high, for example during active bleeding or some types of thrombocytopenia.

## Introduction

The relative contributions of intrinsic versus extrinsic factors in regulating platelet life span have been the subject of considerable debate and controversy. A popular explanation first articulated by Mustard, Roswall and Murphy favoured the extrinsic viewpoint [1]. Their “multiple-hit” model holds that platelets age by accumulating damage over time, for example by temporary involvement in thrombi or via stress, shear or temperature fluctuations during normal circulation [1], [2]. Once a critical level of damage is reached, the platelet is cleared by the reticuloendothelial system. Studies of size, density and morphology as platelets age appeared to support this model [3]–[11]. However, recent molecular data have challenged this primarily extrinsic view of platelet ageing. Both platelet count and platelet turnover are altered in mice with deficiencies in pro- and anti-apoptotic proteins (in particular Bak and Bcl-x_L_), indicating an essential role for the intrinsic apoptosis pathway in platelet homeostasis [12].

We recently attempted to integrate this new molecular information with the multiple-hit model by fitting the latter to population and cohort survival curves from mice with mutations in Bcl-x_L_ and Bak [13]. Although the multiple-hit model generated adequate fits to the data, the results suggested that it was primarily the rate of hits rather than the number of hits that differed between the genotypes – a result that is difficult to reconcile with the original exposition of the model. Thus, a simpler interpretation was that, under normal physiological conditions, platelet life span is not regulated by damage inflicted by extrinsic “hits”, but instead is programmed by an internal timer. We proposed that intrinsic factors (such as the levels of pro- and anti-apoptotic proteins) would give rise to a natural distribution of platelet life span. Although many choices for the mathematical form of this distribution were possible, we chose the lognormal distribution as it appeared to provide a simple explanation of the differences between genotype (i.e. shifting mean log life span). We refer to the resulting mathematical model as the “lognormal-senescent” or LS model.

Our conclusion that platelet life span is internally regulated left open the questions of how the apoptotic program in platelets is triggered, and what mechanism determines the point at which each individual platelet enters the apoptotic process. Furthermore, our modelling did not account for the fraction of platelets that are consumed by normal hemostatic requirements. Hanson *et al.* demonstrated a fixed requirement for platelets by studying platelet survival in patients with varying degrees of bone marrow hypoplasia [14]. They used a novel approach, first extracting an estimate of mean life span from the gamma-fit (multiple-hit) method, and then using the Dornhorst model to correlate mean life span and platelet count across all patients, assuming an equal and fixed requirement for platelets in each individual. The Dornhorst model is a classic model incorporating senescent death at a fixed time with random loss up until that time. It was described in 1951 for the study of red cell survival curves [15], but can in principle be applied to any cell type with these two alternate cell fates. However, the model suffers from the problem that it tends to overestimate the random loss fraction when fit directly to individual survival curves. Hanson *et al.*'s approach overcame this problem by not directly relying on fits to survival curves. Rather they focussed on the predicted reduction in both platelet count and life span as platelet production was decreased – an approach made possible by acquiring data from a wide range of patients with differing degrees of hypoplasia, and controls. They concluded that approximately 7,000 platelets per microlitre of blood per day are required to maintain hemostasis, and consequently approximately 18% of platelets in healthy human individuals are consumed before the end of their natural lifespan.

Here, we address the question of whether it is possible to detect the fixed requirement for platelets directly from individual survival curves, i.e. without access to data derived from individuals with variable platelet production rates. The reason that the Dornhorst model tends to overestimate the random loss fraction is that it does not account for the intrinsic variability in cell life span. This approximation works well for human red cells, whose mean life span is approximately 120 days with a standard deviation of no more than 10 days (coefficient of variation (C.V.) <10%). However, for wild-type murine platelets the mean life span is approximately 4 days, with a standard deviation of 1 day (C.V. ∼25%). Hence, the curved “tail” of the survival curve tends to result in a spuriously high estimate of the random loss fraction when the Dornhorst model is fitted. We therefore developed a hybrid of the Dornhorst model and the LS model introduced in our previous study, which we refer to as the Dornhorst-LS or DLS model. Our results are consistent with a fixed requirement for platelets to maintain hemostasis, however the biological interpretation of our results is limited by poor constraint of the critical parameter related to random loss. We quantitatively compare humans and mice, and note that on face value our results suggest a fixed requirement for platelets in mice of the order of 100,000 per microliter of blood per day, much higher than the corresponding value in humans. We then explore some of the technical difficulties with our approach that contribute to poor parameter constraint. Overall, our data and analysis suggest that at steady-state there is a large excess of platelets and therefore the random loss fraction is small, and difficult to detect by model fitting to survival curves alone.

## Results and Discussion

Recently, we developed an *in vivo* double-labeling technique for studying platelet survival. First, the X488 reagent (Emfret Analytics, Eibelstadt, Germany) is injected intravenously to label the majority of platelets in circulation at that particular time, thus establishing a “population label”. X488 is a DyLight488-labeled rat IgG derivate against the murine GPIbβ subunit of the platelet specific GPIb-V-IX complex. Subsequently (24 hr in our study) the standard technique of *in vivo* biotinylation is performed [16]. Platelets that are negative for the first label but positive for the second represent a “cohort” of platelets born in the time period between the two labelings. Flow cytometric analysis to enumerate the percentage of platelets carrying combinations of the two labels yields population and cohort survival curves.

In our previous study we fit two different models, the multiple-hit model and the LS model, to population and cohort data in wild-type, *Bcl-x^+/Plt20^* and *Bak^−/−^* mutant mice (for which platelet life span is shortened or lengthened, respectively). Both models explain senescent age-dependent platelet death (via different mechanisms) and appeared to provide adequate fits to the data. In this study, we address the question of age-independent random loss (e.g. by consumption in blood clots) and whether its effect on survival curves can be detected.

As discussed in **Materials and Methods**, parameter constraint is an important issue in addressing this question. To this end, we employed a Monte Carlo technique to estimate confidence intervals. Briefly, this involves first modelling the experimental uncertainty about the mean of the data as normally-distributed (Gaussian) noise. A new, simulated set of survival curves is then generated with Gaussian noise of the correct magnitude added about the mean. The model is then refit to this simulated data and a new set of parameters obtained. This process is repeated 1000 times, and the empirical distribution of parameters obtained is used to estimate confidence intervals in those parameters – indicated by box-and-whisker plots with outliers (outside of 2.5–97.5 percentiles) plotted individually as dots in the figures of this paper.

As well as the intrinsic parameters of the models considered here (mean life span, standard deviation of life span, and random loss rate constant) additional parameters are required to fit to the experimental data. In particular, because the labels are not perfect (i.e. do not label 100% of platelets) parameters representing the efficiencies of the two labels, *e_1_* and *e_2_*, are required. Furthermore, as discussed previously [13], the second label – biotin – tends to continue to label platelets for some period of time after the initial injection and so another parameter for its half-life is required, *b_1/2_*. These extra parameters are potential confounders in interpreting our results, so it is best to minimise their impact. To fit the population survival data requires only one of these additional parameters, *e_1_*, whereas to fit the cohort survival data requires the other two, *e_2_* and *b_1/2_*, as well. Therefore, any advantage that may be gained by fitting the cohort data must be balanced against the complication of two additional parameters. We found that fitting the cohort data did not provide any improvement in the constraint of the parameters of interest, therefore in the main text we present results from fitting just the population survival data. In **Supplementary Figures and Tables** we present the results from fitting to both the population and cohort survival data, for reference.

### Lognormal-Senescent (LS) model

**Figure 1:**
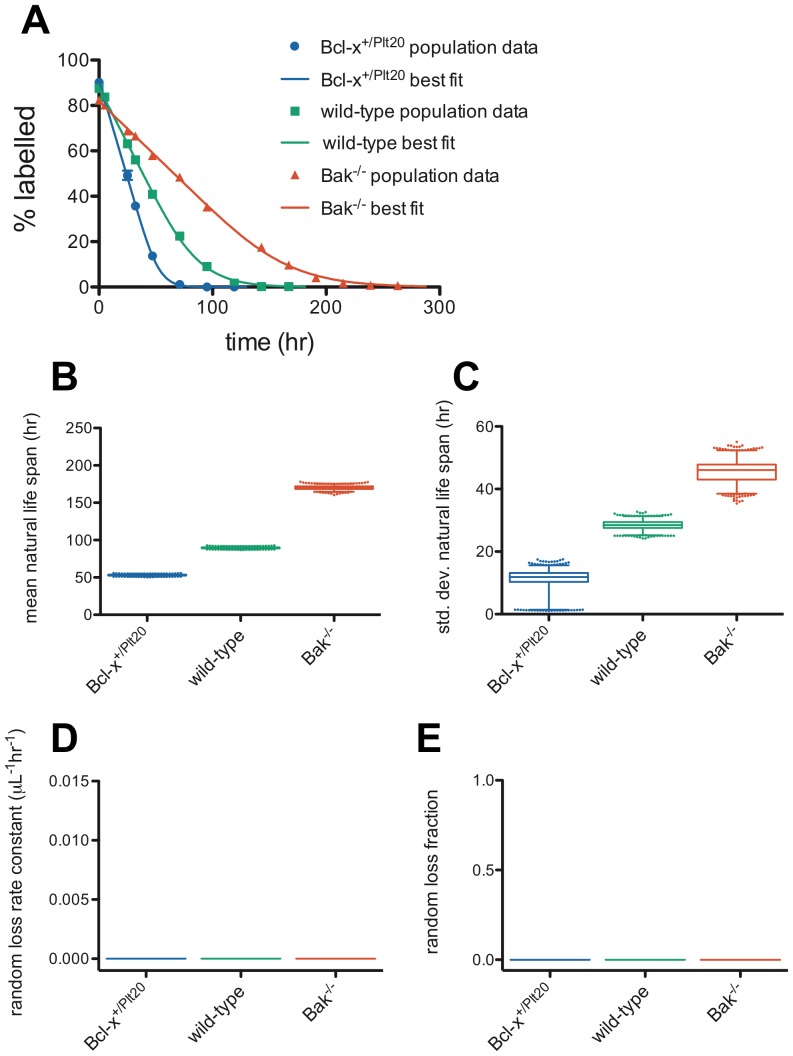
Lognormal-Senescent model fits of platelet survival data. (**A**) Population survival data and LS model best fits for *Bcl-x^+/Plt20^* (blue), wild-type (green) and *Bak^−/−^* (red) mice. A Monte Carlo technique was used to generate estimates of confidence intervals for the model parameters – (**B**) mean natural life span, *μ*, (**C**) standard deviation of natural life span, *σ*, (**D**) random loss rate constant, *r* (always 0 hr^−1^ for this model), and (**E**) random loss fraction, *f* (always 0 for this model). 1000 Monte Carlo simulations were performed and fit to obtain parameters – box-and-whisker plots indicate median, interquartile range, 2.5 and 97.5 percentiles, and outliers are plotted as individual dots.

**Figure 2:**
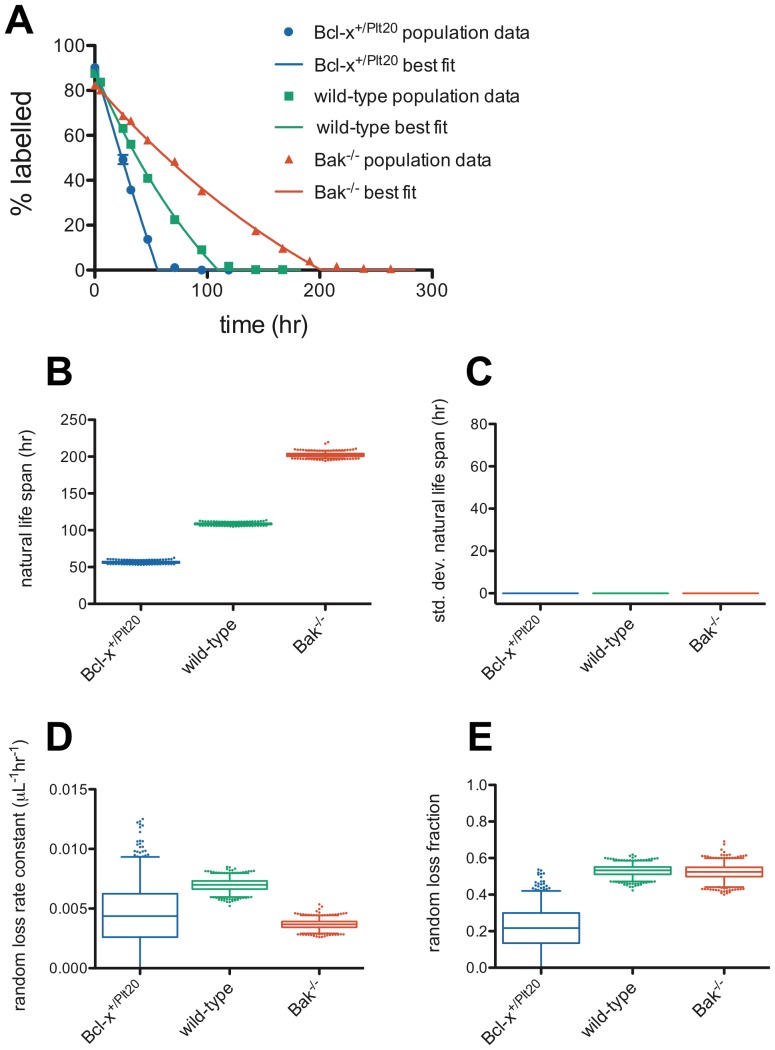
Dornhorst model fits of platelet survival data predict that a large proportion of platelets are destroyed randomly. (**A**) Population survival data and Dornhorst model best fits for *Bcl-x^+/Plt20^* (blue), wild-type (green) and *Bak^−/−^* (red) mice. A Monte Carlo technique was used to generate estimates of confidence intervals for the model parameters – (**B**) natural life span, *T*, (**C**) standard deviation of natural life span (always 0 hr for this model), (**D**) random loss rate constant, *r*, and (**E**) random loss fraction, *f*. 1000 Monte Carlo simulations were performed and fit to obtain parameters – box-and-whisker plots indicate median, interquartile range, 2.5 and 97.5 percentiles, and outliers are plotted as individual dots.

**Figure 3:**
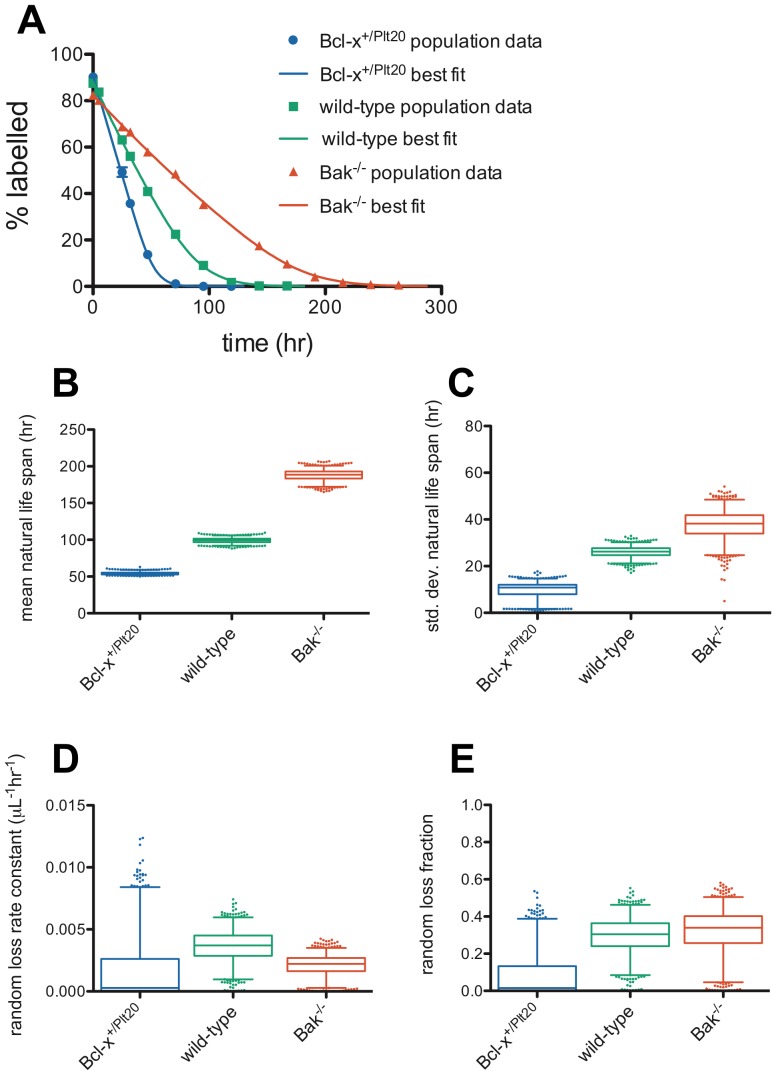
Dornhorst-Lognormal-Senescent model fits of platelet survival data reveals a smaller random loss fraction than the classic Dornhorst model but wide confidence intervals. (**A**) Population survival data and DLS model best fits for *Bcl-x^+/Plt20^* (blue), wild-type (green) and *Bak^−/−^* (red) mice. A Monte Carlo technique was used to generate estimates of confidence intervals for the model parameters – (**B**) natural life span, *T*, (**C**) standard deviation of natural life span, (**D**) random loss rate constant, *r*, and (**E**) random loss fraction, *f*. 1000 Monte Carlo simulations were performed and fit to obtain parameters – box-and-whisker plots indicate median, interquartile range, 2.5 and 97.5 percentiles, and outliers are plotted as individual dots.

We began by reproducing the results of our earlier work [13], [17], fitting the LS model using the slightly altered methodology described above. Results are illustrated in Figure 1 and best-fit parameter values and 95% confidence intervals are reported in Table 1. In Figure 1A, we show what visually appear to be good fits to the population survival curves for the three genotypes. Figures 1B and C show confidence intervals for the mean and standard deviation parameters, *μ* and *σ*, respectively, from the Monte Carlo simulation. Figure S1A and B, illustrate the same information with the alternate parameters –the mean log life span, *m*, and the standard deviation of log life span, *s* – showing that it is primarily the mean log life span, not the standard deviation in log life span, that varies between genotypes, in agreement with our earlier study [13]. Figure 1D and E emphasise that there is no random loss in this model, and are included simply for comparison to the Dornhorst and Dornhorst Lognormal-Senescent models in Figures 2 and 3, respectively.

**Table 1:** Platelet counts (±standard error of the mean, s.e.m.) and best-fit LS model parameters from fits to population survival data for each genotype, with 95% C.I.'s from the Monte Carlo technique in brackets.

genotype	Platelet count, *N* (×10^3^ µL^−1^)	Production rate, *S* (×10^3^ µL^−1^ hr^−1^)	mean life span, *μ* (hr)	std. dev. life span, *σ* (hr)	mean log life span, *μ*	std. dev. log life span, *σ*	labeling efficiency, *e_1_*
*Bcl-x^+/Plt20^*	847±35	15.9 [15.4,16.5]	53.3 [51.2,55.0]	11.3 [1.4,15.6]	3.95 [3.89,4.00]	0.209 [0.026,0.294]	0.910 [0.894,0.925]
wild-type	1183±70	13.2 [12.9,13.5]	89.5 [87.6,91.7]	28.2 [25.3,31.3]	4.45 [4.42,4.48]	0.307 [0.273,0.344]	0.879 [0.871,0.886]
*Bak^−/−^*	1798±148	10.6 [10.3,10.9]	169.5 [164.7,175.3]	47.0 [38.4,52.3]	5.10 [5.06,5.14]	0.272 [0.217,0.307]	0.819 [0.808,0.828]

### Dornhorst model

To assess the possibility of age-independent random loss in addition to the usual age-dependent senescent clearance, we next fit the classic Dornhorst model [15] to our population survival data across the three genotypes. The Dornhorst model assumes that senescent death happens at a definite, fixed age, and that prior to senescent death cells are destroyed (or consumed) randomly at a fixed rate. Thus, there are two parameters of the model in its pure form – the fixed natural life span, *T*, and the random loss rate constant, *r*, as well as the labeling efficiency parameter, *e_1_*. The relative proportion of platelets that are randomly lost or undergo age-dependent senescent clearance is of interest in discussions of platelet fate. We refer to the proportion of cells that are randomly lost (prior to the end of their natural life span) as the “random loss fraction”, *f*, throughout this paper. At steady-state in normal individuals the major source of this random loss of platelets is envisaged to be hemostatic consumption, and thus the random loss rate constant and fraction could equally well be referred to as “consumption rate constant” and “consumption fraction”. Equally, in situations where random destruction of platelets is increased, such as immune thrombocytopenia purpura, “destruction” may be a more appropriate adjective than “loss”. However, we use the more general term “random loss” throughout our discussion, to emphasise the more general applicability of the model. In the Dornhorst model, the random loss fraction is dependent on the random loss rate constant, *r*, and the natural life span, *T*, via Equation (8) in **Materials and Methods**.

Figure 2 illustrates Dornhorst model fits to our data, with confidence intervals on parameter values using the Monte Carlo technique as described above. Best-fit parameter values and 95% confidence intervals are shown in Table 2. As expected, the natural life span is different in the three genotypes (Figure 2B). More surprisingly, the Dornhorst model predicts very high values of the random loss rate constant and fraction (Figure 2D and E) – suggesting of the order of 50% of platelets are randomly lost in wild-type and *Bak^−/−^* mice (and that this proportion is well-constrained between approximately 40 and 60%). This is much higher than previous estimates of this quantity in humans [14]. Figure 2C emphasises that in the Dornhorst model natural death occurs at a fixed time (the standard deviation in natural death time is zero). In his original publication, Dornhorst stressed that the assumption of a fixed age of death is an approximation valid when the standard deviation in life span is small compared with the mean, as is the case for human red cells [15]. As discussed above, our previous study [13] showed that for murine platelets the standard deviation is actually quite significant (C.V. ∼25%), resulting in a significant “tail” to the population survival curve – the curve tapers out after initially appearing linear. Therefore the approximation of fixed natural life span is unlikely to be appropriate for this system. The only way for the Dornhorst model to fit to the tail region of the population survival curve is by increasing the random loss fraction. Therefore, we wondered whether the apparently high values for the random loss estimated by the Dornhorst model were, in fact, an artefact of assuming a fixed death time.

**Table 2:** Platelet counts (±s.e.m.) and best-fit Dornhorst model parameters from fits to population survival data for each genotype, with 95% C.I.'s from the Monte Carlo technique in brackets.

genotype	Platelet count, *N* (×10^3^ µL^−1^)	Production rate, *S* (×10^3^ µL^−1^ hr^−1^)	Natural life span, *T* (hr)	random loss rate constant, *r* (µL^−1^ hr^−1^)	random loss rate, *R* (µL^−1^ day^−1^)	random loss fraction, *f*	labeling efficiency, *e_1_*
*Bcl-x^+/Plt20^*	847±35	16.4 [15.4,18.7]	55.8 [54.0,59.4]	0.0029 [0.0000,0.0092]	59 [0,190]	0.15 [0.00,0.42]	0.911 [0.895,0.928]
wild-type	1183±70	15.6 [14.9,16.1]	109.4 [106.4,111.4]	0.0072 [0.0060,0.0080]	205 [170,226]	0.55 [0.47,0.59]	0.888 [0.880,0.896]
*Bak^−/−^*	1798±148	12.5 [11.9,13.3]	201.3 [197.5,207.8]	0.0035 [0.0029,0.0044]	153 [124,190]	0.51 [0.44,0.60]	0.830 [0.819,0.842]

### Dornhorst Lognormal-Senescent (DLS) model

To address the issue of artefactually high values of the random loss fraction with the Dornhorst model, we developed a model, which we refer to as the “Dornhorst Lognormal-Senescent” model (or DLS model) see **Materials and Methods**, where senescent death occurs according to a lognormal distribution, rather than at a fixed time, and random death occurs independently – potentially cutting short the natural life span. Thus the DLS model has three parameters – the mean, *μ*, and standard deviation, *σ*, of the lognormal distribution of senescent life spans, and the rate constant for random loss of platelets, *r* (as well as the additional parameter for the labeling efficiency, *e_1_*, needed to fit real data). The random loss fraction, *f*, in this model depends on the distribution of natural life spans, *L(⋅)*, and the random loss rate constant, *r*, via Equation (12) in **Materials and Methods**. The choice of a lognormal distribution for natural lifespan is not essential, and similar results can be obtained with other positive, right-skewed distributions such as the gamma distribution.

Figure 3 illustrates the results of fitting the DLS model to the population survival data across the three genotypes, and Table 3 reports best-fit parameter values and 95% confidence intervals. Figures 3B and C show the expected result of increasing mean life span and standard deviation of life span across the genotypes, and Figures S1C and D show that the result of increasing mean log life span while standard deviation log lifespan remains constant (within parameter constraints), seen in the LS model, is also true in the DLS model. More importantly, as can be seen in Figure 3E, this model does appear to allow for much lower value of the random loss fraction when compared with the Dornhorst model, Figure 2E. In *Bcl-x^+/Plt20^* there is little evidence for random loss (best-fit *f* = 0.00, 95% C.I. [0.00,0.39]), while in wild-type (best-fit *f* = 0.31, 95% C.I. [0.09,0.46]) and *Bak^−/−^* (best-fit *f* = 0.34, 95% C.I. [0.04,0.51]). Thus, while the best-fit value may still seem rather high in wild-type and *Bak^−/−^*, the confidence intervals are very wide and therefore it would be inappropriate to draw strong conclusions regarding the value of this parameter and its biological significance.

**Table 3:** Platelet counts (±s.e.m) and best-fit DLS model parameters from fits to population survival data for each genotype, with 95% C.I.'s from the Monte Carlo technique in brackets.

genotype	Platelet count, *N* (×10^3^ µL^−1^)	Production rate, *S* (×10^3^ µL^−1^ hr^−1^)	mean life span, *μ* (hr)	std. dev. life span, *σ* (hr)	mean log life span, *μ*	std. dev. log life span, *σ*	random loss rate constant, *r* (µL^−1^ hr^−1^)	random loss rate, *R* (µL^−1^ day^−1^)	random loss fraction, *f*	labeling efficiency, *e_1_*
*Bcl-x^+/Plt20^*	847±35	16.0 [15.6,19.2]	53.3 [51.3,59.0]	11.3 [1.7,14.7]	3.95 [3.91,4.07]	0.209 [0.030,0.274]	0.0003 [0.0000,0.0083]	6 [0,169]	0.02 [0.00,0.39]	0.909 [0.895,0.927]
wild-type	1183±70	14.4 [13.5,15.3]	99.5 [92.1,105.8]	26.1 [21.2,30.1]	4.57 [4.48,4.64]	0.258 [0.202,0.313]	0.0038 [0.0011,0.0060]	108 [30,169]	0.31 [0.09,0.46]	0.883 [0.875,0.892]
*Bak^−/−^*	1798±148	11.8 [10.6,12.7]	189.0 [172.1,200.9]	38.1 [24.7,48.2]	5.22 [5.12,5.29]	0.200 [0.124,0.270]	0.0023 [0.0002,0.0035]	97 [11,151]	0.34 [0.04,0.50]	0.826 [0.814,0.837]

The DLS model can be viewed in two ways – (1) as an extension of the LS model to include a random loss term, or (2) as an extension of the Dornhorst model to allow a non-zero standard deviation in the distribution of natural lifespan (the choice of distribution is obviously not unique, but here we work with the lognormal distribution). Either way, there is one extra parameter in the more complex model (the DLS model) compared with the simpler model (LS or Dornhorst) and the simpler model is said to be “nested” in the more complex model. The F-test is a statistical test that can be used in circumstances such as these (nested models), to say whether the addition of extra parameters with the more complex model is justified by the improvement in quality of fit [18]. The test as it applies here is described in **Materials and Methods**. The results of an F-test comparing the DLS to the LS model are shown in Table 4, and comparing the DLS to the Dornhorst model in Table 5. The F-test can be viewed as a type of statistical hypothesis test, with the simpler model (LS or Dornhorst) considered the null hypothesis, and the more complex model (DLS) considered the alternate hypothesis. The relevant quantities for each fit are the sum of square residuals (*s.s.r.*) and the number of degrees of freedom (*df*), which is equal to the number of data points minus the number of parameter values. The F-test shows the improvements in quality of fit were statistical significant for both model comparisons in the wild-type and *Bak^−/−^* genotypes, but not for either model comparison in the *Bcl-x^+/Plt20^* genotype.

**Table 4:** F-test to compare best fits with the LS model (the null hypothesis) versus the DLS model (one extra parameter – the alternative hypothesis).

	*#data points*	*#params_null_*	*df_null_*	*#params_alt_*	*df_alt_*	*ssr_null_*	*ssr_alt_*	*F*	*P*
*Bcl-x^+/Plt20^*	48	3	45	4	44	295.2	295.2	0.00	1
wild-type	60	3	57	4	56	106.7	95.2	6.77	0.011
*Bak^−/−^*	78	3	75	4	74	314.5	290.5	6.11	0.016

**Table 5:** F-test to compare best fits with the Dornhorst model (the null hypothesis) versus the DLS model (one extra parameter – the alternative hypothesis).

	*#data points*	*#params_null_*	*df_null_*	*#params_alt_*	*df_alt_*	*ssr_null_*	*ssr_alt_*	*F*	*P*
*Bcl-x^+/Plt20^*	48	3	45	4	44	314.4	295.2	2.86	0.098
wild-type	60	3	57	4	56	140.2	95.2	26.5	3e-6
*Bak^−/−^*	78	3	75	4	74	335.2	290.5	11.37	0.0012

This improvement in quality of fit as assessed by the F-test could be taken as evidence the DLS model is superior to either the LS or Dornhorst models, at least in the wild-type and *Bak^−/−^* genotypes. However, this result must be interpreted with caution. As noted above, the wide confidence intervals for the random loss parameter limit the usefulness of the results. For example, no firm conclusion can be drawn as to how the random loss fraction varies, if at all, between the three genotypes studied here.

### Fitting to the cohort survival data as well as the population survival data does not substantially improve confidence intervals

Tables S1, S2, S3 show the results of fitting each of the models – the LS, Dornhorst, and DLS models – to both the population and cohort survival data for each of the three genotypes. As discussed above, to fit the cohort survival data necessitates the introduction of two additional parameters – the labeling efficiency for the second label, *e_2_*, and the biotin half-life, *b_1/2_*. As can be seen from comparing the confidence intervals in these tables to those in Tables 1, 2, 3, there is very little improvement (narrowing) of the confidence intervals for the parameters, in particular the random loss fraction, *f*, of most interest in this paper. Thus the cohort survival data does not help significantly in constraining parameter values. As an illustration of the fits that can be obtained to the double-labeling data, Figure S2 shows fits using the DLS model for each of the genotypes, with the corresponding best-fit parameter values and 95% confidence intervals are reported in Table S3. Tables S1 and S2 report the best-fit parameter values and 95% confidence intervals for fits to the double-labelling data with the LS and the Dornhorst models, respectively.

### Comparison between mice and humans

Some quantitative comparisons between mice and humans are insightful at this point. At steady-state production of platelets (*S*) must balance their clearance/loss, therefore a rough estimate of the production rate is the total platelet count (*N*) divided by the mean life span of platelets (*μ*), *S≈N/μ*. Typical values of these quantities in humans are approximately *N* = 250×10^3^/µL, *μ* = 9 days, and so *S*≈28×10^3^/µL/day. In wild-type C57BL/6 mice, approximately *N* = 1200×10^3^/µL, *μ* = 4 days, and so *S*≈300×10^3^/µL/day. Therefore mice have a roughly 10-fold higher production/turnover of platelets per microliter of blood per day, compared with humans.

Furthermore, according to Equation (15) in **Materials and Methods**, the random loss fraction equals the absolute random loss rate divided by the production rate, *f = R/S*. The absolute random loss rate is the random loss rate constant times by the platelet count, *R = rN*, and is equivalent to Hanson *et al.*'s “fixed requirement for platelets” [14]. Hanson *et al.* estimated the fixed requirement for platelets in humans to be 7×10^3^/µL/day. Combining this value with the estimate of the production rate above, gives *f*≈0.25, in reasonable agreement with what Hanson *et al.* reported (*f*≈0.18) given the approximate nature of these calculations. The fixed requirement for platelets in mice is currently unknown, however if it is the same as in humans, Equation (15) would imply that the random loss fraction would only be *f*≈0.02, due largely to the much higher production rate in mice.

**Figure 4:**
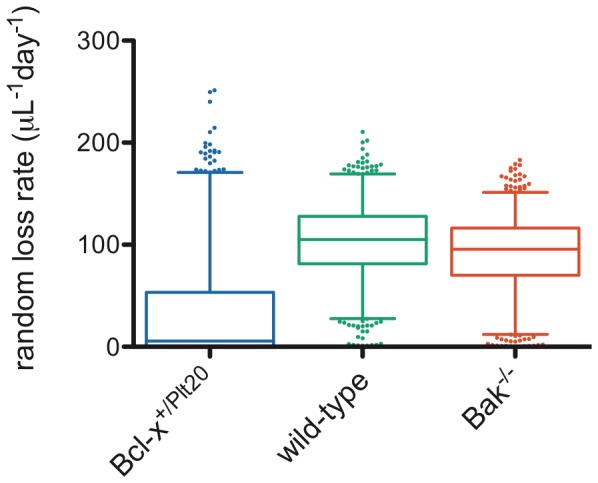
Values of absolute random loss rate (fixed requirement for platelets), extracted from the same fits of the DLS model to survival data as illustrated in **Figure 3**. 1000 Monte Carlo simulations were performed and fit to obtain parameters – box-and-whisker plots indicate median, interquartile range, 2.5 and 97.5 percentiles, and outliers are plotted as individual dots.

Of course, these calculations are only estimates, however they give some insight into the expected value of the random loss fraction if the fixed requirement for platelets is the same between mice and humans. For technical reasons discussed further below, such a low value of the random loss fraction (*f*≈0.02) would be very difficult to detect in the survival curves. By contrast, the higher values of the random loss fraction reported in Figure 3 (*f*≈0.35) can be seen to correspond to a much higher fixed requirement for platelets. Figure 4 shows the fixed requirement for platelets, expressed per microliter of blood per day for ease of comparison to Hanson *et al.*'s results. As can be seen, in wild-type and *Bak^−/−^* the fits would suggest a fixed requirement for platelets of closer to 100×10^3^/µL/day, whereas in *Bcl-x^+/Plt20^* the fixed requirement was not convincingly detectable.

Several resolutions are possible. Our results may be correct and mice may indeed have a fixed requirement for platelets of around 100×10^3^/µL/day, much higher than the equivalent figure in humans of 7×10^3^/µL/day. The fact that we could not detect this fixed requirement in the *Bcl-x^+/Plt20^* genotype might be explained by the fewer data points on those survival curves making it difficult to detect the precise curvature. If the difference between species is indeed real, consideration of other quantitative differences in mouse versus human physiology (such as body weight, blood volume, platelet size and surface area of vessels) may prove insightful. Alternatively, our results are misleading due to technical difficulties in extracting the value of the random loss fraction from the survival curves. We therefore decided to explore some of the technical difficulties associated with fitting the model to data, and the implications for the interpretation of our results.

### Trade-off between mean life span and random loss fraction in the DLS model

**Figure 5:**
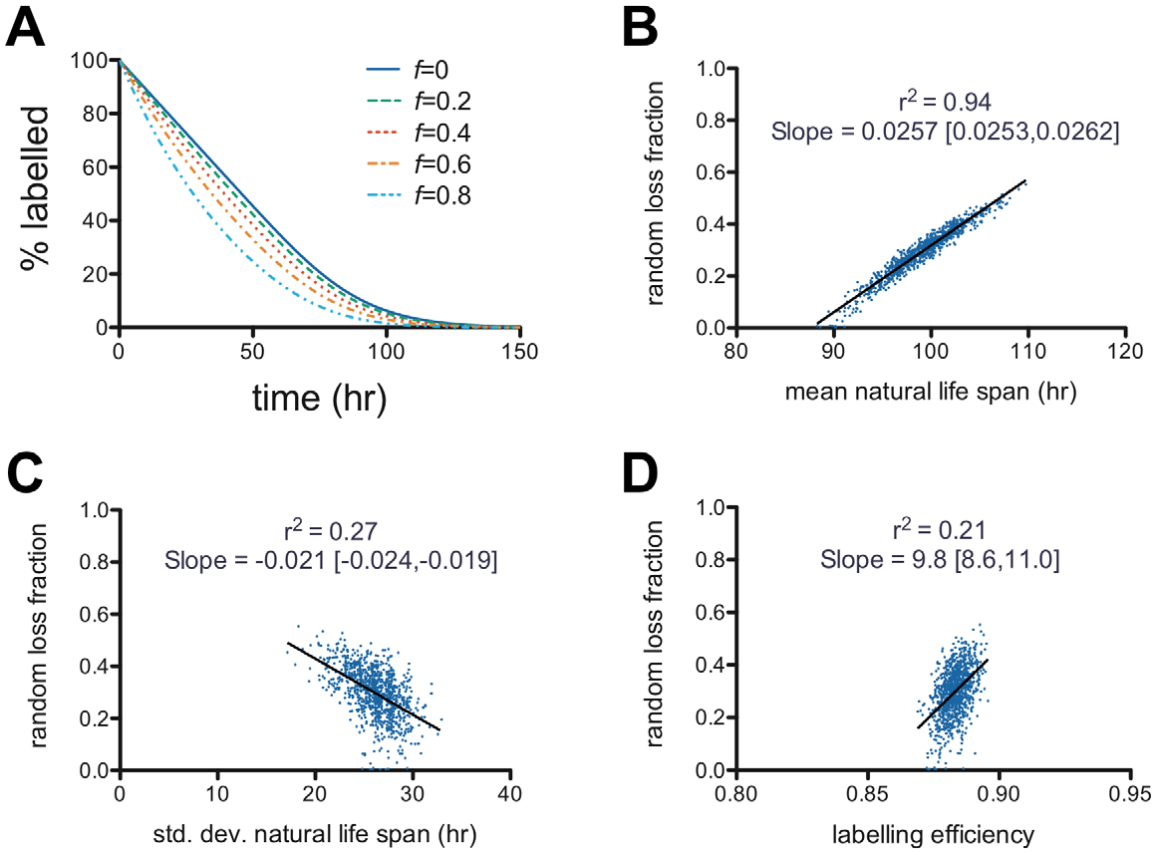
Illustration of the difficulty in constraining the value of the random loss fraction using the Dornhorst-Lognormal-Senescent model. (**A**) Theoretical population survival curves from the DLS model as the mean and standard deviation of natural life span are kept constant (*μ* = 100 hr, *σ* = 25 hr) while the random loss fraction, *f*, is varied. Small values of the random loss fraction produce subtle changes in the shape of the population survival curve. (**B**) Strong correlation between values of the random loss fraction (*f*) and the mean natural life span (*μ*) in the Monte Carlo simulation – r^2^ is the square of Pearson's correlation coefficient; Slope is the gradient of the linear regression with 95% confidence intervals in brackets [,]. The correlation with other parameters of the model is much less – (**B**) random loss fraction (*f*) versus standard deviation natural life span (*σ*), and (**C**) random loss fraction (*f*) versus labeling efficiency (*e_1_*).

The poor constraint on the random loss fraction revealed by the Monte Carlo simulation warranted further investigation. Part of the difficulty appears to stem from the fact that small values of the random loss fraction have only very subtle effects on the predicted survival curves. Figure 5A illustrates this point. Here we have generated theoretical predictions for the population survival curve with the DLS model by keeping the natural life span distribution constant while the random loss fraction is varied. As long as the random loss fraction is relatively small (say, 0.4 and below in the figure) the survival curve takes a qualitatively very similar shape. It looks linear initially, and tapers out to a tail around the mean natural life span. It would be very difficult by eye to distinguish the effect of the increasing the random loss fraction from shortening the mean natural life span. It is only when the random loss fraction is relatively large (say, 0.6 and above in the figure) that the initial segment of the survival curve starts to take a noticeably non-linear shape due to the contribution of random loss. The trade-off between the mean life span parameter and the random loss fraction is further illustrated in Figure 5B, which is a scatter-plot of mean life span versus random loss fraction for the wild-type genotype from the Monte Carlo simulation. Linear regression was performed and Pearson's correlation co-efficient was calculated, which showed a high correlation between these two parameter values (*r^2^* = 0.94). This result further illustrates the point that the poor constraint on the random loss fraction is largely due to a trade-off with the mean life span – namely, a small increase in both simultaneously can produce an almost identical initial slope to the population survival curve. For completeness, Figures 5C and D show that there is much weaker correlation of the random loss fraction with other parameters of the model – the standard deviation in life span and the labeling efficiency. Similarly, Figure S3 shows little pair-wise correlation amongst the other parameters of the model.

### Relationship between parameter constraint and experimental uncertainty

**Figure 6:**
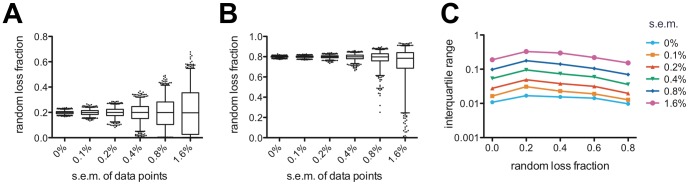
The effect of experimental uncertainty on the ability to constrain the value of the random loss fraction. Fits to simulated data with Gaussian random noise of varying standard error of the mean (s.e.m.) added to simulate experimental uncertainty. (A)–(E) Simulated model parameters: *μ* = 100 hr, *σ* = 25 hr, with (A) *f* = 0.2, (B) *f* = 0.8. For each value of *f* and each value of the s.e.m. of the noise, the fit was repeated 1000 times with independently-generated noise – the box-and-whisker plots indicate median, interquartile range, 2.5 and 97.5 percentiles, and outliers are plotted as individual dots. (C) Interquartile range as a function of random loss fraction for the various levels of noise added to the simulated data. For a given value of the s.e.m. of the noise, higher values of the random loss fraction are generally better constrained than lower (non-zero) values.

Finally, we attempted to quantify the relationship between experimental uncertainty and parameter constraint in this system. In practice it is impossible to eliminate experimental uncertainly entirely or to vary its degree. Therefore, to answer this question we decided to fit to simulated data generated directly from the model with varying degrees of random noise added. We chose to fix the mean and standard deviation at μ = 100 hr, σ = 25 hr, and vary the random loss fraction over a range of values, *f* = 0, 0.2, 0.4, 0.6, and 0.8. For each value of *f* we generated baseline “ideal” data. Experimental uncertainty was modelled as Gaussian noise with a mean of 0 and a standard deviation of varying magnitude. For each value of the noise we simulated a single data set and fit the DLS model to it, followed by the Monte Carlo technique to estimate confidence intervals, exactly as was done for the real data. The resulting confidence intervals are shown in Figure 6, (A) *f* = 0.2, (B) *f* = 0.8, representative of low and high random loss fractions, respectively. For all values of *f*, as the value of the random noise is increased the confidence intervals rapidly widen. We note, however, that for higher values of *f* the confidence intervals tend to be narrower for any given value of the noise. This point is illustrated in Figure 6C which plots the interquartile range as a function of *f* for different values of the noise. Roughly, at *f* = 0.8 an equivalent interquartile range can be achieved with twice as much noise compared with *f* = 0.2.

To be able to draw useful conclusions as to how the random loss fraction varies between individuals or in different disease states, one requires relatively narrow confidence intervals around the parameter values. One can see from Figure 6 that reasonable confidence intervals are only achieved at the lower values of the random noise, say s.e.m. = 0.2% and below. In practice, the implication is that the standard error in the mean (*s.e.m.*) of the data points must be reduced below this threshold to achieve that level of parameter constraint. Theoretically, this can be achieved in two ways – (1) by reducing the experimental uncertainty, or (2) increasing the number of replicates. Usually, the experimental protocol will have been optimised and care taken to minimise as much as possible all potential sources of experimental uncertainty. Therefore, (1) will not usually be possible in practice. The alternative, (2) is to increase the number of replicates. The *s.e.m.* is related to the standard deviation of the data (*s.d.*) and the number of replicates (*n*) by the formula: 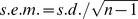. As an example, for our wild-type data, *s.d.* = 1.26%, *n* = 6, and therefore *s.e.m.* = 0.56%. Assuming the *s.d.* cannot be improved, we would need *n*>41 for *s.e.m.*<0.2%, and *n*>160 in order for *s.e.m.*<0.1%. Thus the task of collecting sufficient experimental replicates is clearly onerous and may be prohibitive in many circumstances. However, as noted above, experimental uncertainty is somewhat more tolerable at higher values of the random loss fraction; thus the model is likely to find greater utility in disease states where higher values of the random loss fraction might be expected.

## Conclusions

Putting aside the issue of parameter constraint and differences between mice and humans, our results are at least consistent with the view that in steady-state the number of platelets is far in excess of the body's usual requirements and suggest an attractive resolution to the debate over the relative importance of internal and external processes in regulating platelet life span. Combined with our previous results [13], we hypothesise that the default state is for platelets to initiate apoptosis as they exhaust their natural life span. This process is truly internal to the platelets and would occur even if they were shielded from any form of environmental damage. However, environmental factors, such as consumption in blood clots or other forms of damage, lead to loss of some platelets from the circulation before their natural life span is exhausted. The proportion of platelets lost in this random way appears to be low in healthy individuals, but is likely to be higher in disease states such as certain thrombocytopenias or atherosclerosis, and with advanced age, where survival curves look more curvilinear [19]–[21].

Finally, we note that the model introduced here can be used to study any cell population where there are two alternate fates – age-dependent clearance according to a defined probability distribution (e.g. intrinsic senescent death), and random loss (e.g. due to some extrinsic process that consumes or destroys cells indiscriminately). This paradigm of competing intrinsic and extrinsic effects on cell fate is found broadly in cell biology *in vivo*, thus our model may prove useful in the analysis of other cell systems where quantification of these two alternate fates is desired.

## Materials and Methods

### Ethics statement

All animal experiments complied with the regulatory standards of, and were approved by, the Walter and Eliza Hall Institute Animal Ethics Committee.

### Mice

Mice with the *Plt20* mutation in *Bcl-x* [12] and *Bak^−/−^* mice [22] have been previously described. Both mutations had been backcrossed at least 10 generations to C57BL/6 background. Wild-type controls were C57BL/6.

### Experimental techniques

The experimental techniques for *in vivo* double labeling with DyLight488 conjugated NHS-biotin and subsequent flow-cytometric analysis to obtain population and cohort survival, have been described previously [13]. Double labeling was performed in 6 mice of each genotype: *Bcl-x^+/Plt20^*, wild-type and *Bak^−/−^*.

### Mathematical modeling

Mathematical modeling of a cell population in steady-state where the distribution of life spans is fixed and known, has been described previously [13], [15], [23]. We briefly review the main points here to establish notation as a basis for discussing the Dornhorst and DLS models. Let *L(l)* be the age-dependent distribution of life spans, *l*. The probability, *P(a)*, of surviving to age *a* is:
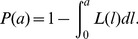
(1)At steady-state, the number of platelets of age *a* must be proportional to the probability of surviving to that age. Therefore, the (normalised) probability density of ages in the population is:
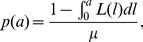
(2)where 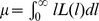 is the mean life span. Because this is a steady-state condition, after time *t* those platelets of age less than *t* will be new. Therefore, the survival curve *D(t)* takes the form:
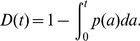
(3)When fitting to population survival data, this theoretical survival curve must be scaled by the efficiency of labeling with the first label, *e_1_* (typically approximately 0.9 for the X488 label in our experiments), which is an additional parameter in the fitting. Finally, assuming the total blood volume is constant, and platelet production occurs at a constant rate, *S*, then the number of platelets, *N*, is predicted to be:
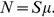
(4)Platelet counts and all rates (of production and destruction) are measured per microliter of total blood throughout this paper.

### Lognormal-senescent (LS) model

In this model the life span distribution is lognormal:
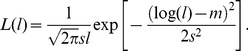
(5)The parameters *m* and *s* are the mean and standard deviation, respectively, of the logarithm of life span. When the life span distribution is lognormal, the survival curve always has an analytic form [17].

### Dornhorst model

The Dornhorst model was original developed to interpret red cell survival curves [15]. Briefly, cells (in this case platelets) are assumed to die at a fixed time, *T*, (senescent death) if they are not earlier removed by random loss, which occurs at a constant rate, *r*. The survival curve takes the form:
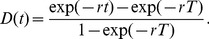
(6)As above, if *S* is the production rate, then the predicted platelet count is:
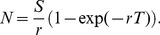
(7)The proportion of platelets that eventually are randomly lost before they die of senescence (the “random loss fraction”, *f*) is:
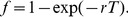
(8)Finally, the mean life span across all platelets is:
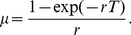
(9)


### Dornhorst-LS model

Here, we derive a model that combines both age-dependent senescent death according to some distribution and age-independent random death at a constant rate, *r*. This can be considered a generalisation of the Dornhorst model where senescent death does not have to occur strictly at some fixed time (*T*) but is distributed.

Following the same logic as above, the probability of surviving to age *a* is:
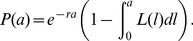
(10)In steady-state, the probability density of cell age in the population must be proportional to this probability, and can be found by normalising this equation:
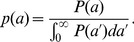
(11)Integration by parts can be used to show that the normalization factor is 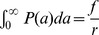, where *f* is the random loss fraction:
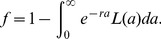
(12)The survival curve can then be calculated as above, Equation (3).

The steady-state platelet number can be derived by requiring that the number of platelets dying at any instant balances the production rate. If *N* is the total number of platelets in the population, then the number dying at any instant is:
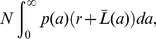
(13)where 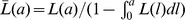 is the rate of natural death at age *a*, given life span distribution *L(⋅)*. Equating this expression with the production rate, *S*, gives (after some working involving integration by parts):
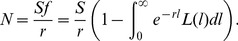
(14)As expected, when *r*→0 this equation reduces to Equation (4) for the case of no destruction (*f/r*→*μ*), and when 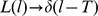 (a delta function representing a fixed life span, *T*) it reduces to Equation (7) for the Dornhorst model.

Finally, rearranging Equation (14) gives:
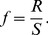
(15)Where we have defined the instantaneous absolute total random loss rate (*R*) as the random loss rate constant (*r*) times by the platelet count (*N*). Thus, the random loss fraction is equal to the ratio of absolute total random loss rate (*R*) to the production rate (*S*). It follows that in situations where random loss is predominantly due to hemostatic consumption, the random loss fraction is a measure of the balance between the requirement for platelets (*R*) and their production (*S*).

When the natural life span distribution is chosen to be lognormal, we refer to this model as the Dornhorst Lognormal-Senescent (DLS) model. When the standard deviation in life span approaches zero, it reduces to the classic Dornhorst model.

### Calculation of predicted cohort label

The double-labeling procedure involves injecting a second label after a delay of time, *d* (24 hrs in our experiments), in order to establish a “cohort”, negative for the first label but positive for the second, that were born within the intervening time. In our experiments the second label is biotin, which is less effective than X488 in labeling platelets (the labeling efficiency, *e_2_*, is approximately 0.6). Ideally, the distribution of ages in the cohort at the time it is established would be truncated at age *d*:
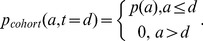
(16)However, in practice we need to account for the labeling efficiencies – thus the initial distribution of ages in the cohort is:
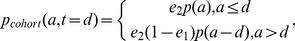
(17)The survival of this population can be calculated by solving a partial differential equation:
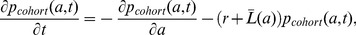
(18)with Equation (16) as the initial condition and with the boundary conditions:
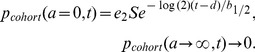
(19)Here, the biotin half-life, *b_1/2_*, has been used to account for the additional labeling of some newly-produced platelets, as described previously [13]. The area under *p_cohort_(a,t)* represents the relative proportion of the initial cohort remaining at time *t*:
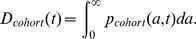
(20)


### Numerical methods

Best fits to survival data were generated by minimizing the sum of square residuals between the data and model predictions using custom code written in MATLAB, as described previously [13], based on the MATLAB function *fmincon* using the ‘interior point’ algorithm. For numerical integration to obtain population and cohort survival curves, the number of grid points was chosen to be 1000. The range for integration was [0,exp(*m*+4*s*)] for the LS or DLS model (where *m* and *s* are the mean and standard deviation of the log of lifespan),or [0,*T*] for the Dornhorst model. To overcome the problem that the best fit may depend on the starting model parameters, we adopted a technique of finding the best fit multiple times from different starting points. Parameter starting points were chosen uniformly and randomly from an interval, and the fit was repeated to see if an improvement to the previous best could be found. Once a better fit had not been found for the previous 100 starting points (population fits) or 10 starting points (population and cohort fits), the optimization was terminated.

The Monte Carlo technique for estimating confidence intervals is slightly different to the bootstrapping technique used in our previous study [13], therefore we describe it here briefly. The method is described in [18]. First a best fit to the data is generated. Next, the experimental error about the model fit is modeled as Guassian noise with a mean of 0 and a standard deviation, *σ_e_*, given by:
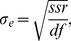
(21)where *ssr* is the sum of square residuals of the best fit, and *df* is the number of degrees of freedom of the data, which is equal to the number of data points minus the number of model parameters. Simulated data is then generated by adding Gaussian noise of this magnitude to each data point on the curve. Since there were 6 replicates in the original experiment we simulate 6 survival curves for each Monte Carlo iteration. A new best fit is then calculated as above. This procedure was repeated for 1000 iterations, thus producing an empirical distribution of parameter values that one might expect to find from repetitions of the experiment. These numerical distributions are used to create the box-and-whisker plots and correlations between parameters in the figures of this paper, and the 95% confidence intervals reported in tables.

### F-test

In general, a more complicated model (one with more parameters) should be expected to produce an improvement in the quality of fit to data (as measured by the sum of square residuals) compared with a simpler model. The F-test [18], [24] can be used to assess the statistical significance of the improvement in fit. First, the F statistic is calculated as:
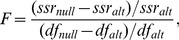
(22)where *ssr_null_* and *ssr_alt_* are the sum of square residuals and *df_null_* and *df_alt_* are the number of degrees of freedom (the number of data points minus the number of model parameters) for the simpler (null hypothesis) and more complicated (alternate hypothesis) models, respectively. Under the null hypothesis that any improvement in fit is due to chance alone, a theoretical distribution of the F statistic (depending on the number of degrees of freedom of the two models) can be calculated. Thus, a p-value can be derived that can be interpreted as the likelihood, by chance alone, that the F statistic takes a value at least as large as is observed. We adopted the conventional threshold value of *p* = 0.05 for statistical significance. Roughly speaking, if the relative improvement in the sum of square residuals is much greater than the relative increase in the number of degrees of freedom, then the result is likely to achieve statistical significance.

The F-test is only valid when the simpler model is a special case of the more complicated model (i.e. the models are nested). In this paper, the classic Dornhorst model and the LS model are both special cases of the DLS model – the Dornhorst model corresponds to the standard deviation of life span being fixed to be zero, and the LS model corresponds to the random loss rate constant being fixed to zero. Therefore, the F-test can be used to assess whether the improvement in the quality of fit with the DLS compared with either the Dornhorst or LS models justifies the inclusion of the extra parameter.

## Supporting Information

Figure S1 Log-transformed parameters of the Lognormal-Senescent and Dornhorst-Lognormal-Senescent model fits. (**A**) and (**B**) Lognormal-Senescent model mean log life span (*m*) and standard deviation log life span (*s*), respectively. (**C**) and (**D**) Dornhorst Lognormal-Senescent model mean log life span (*m*) and standard deviation log life span (*s*), respectively. It is primarily the mean log life span rather than the standard deviation log life span that varies between the genotypes in either model. The three genotypes are identified by colour – *Bcl-x^+/Plt20^* (blue), wild-type (green) and *Bak^−/−^* (red).
(EPS)

Figure S2 Fits of the Dornhorst Lognormal-Senescent model to both population and cohort survival data for the different genotypes. (**A**) *Bcl-x^+/Plt20^*. (**B**) wild-type. (**C**) *Bak^−/−^*.
(EPS)

Figure S3 Weak correlation amongst the parameters of the DLS model other than the random loss fraction. Linear regression of the Monte Carlo parameters for the wild-type genotype – r^2^ is the square of Pearson's correlation coefficient; Slope is the gradient of the linear regression with 95% confidence intervals in brackets [,]. (**A**) mean natural life span (*m*) versus standard deviation natural life span (*σ*). (**B**) labeling efficiency (*e_1_*) versus mean natural life span (*m*). (**C**) labeling efficiency (*e_1_*) versus standard deviation natural life span (*s*).
(EPS)

Table S1 Best-fit LS model parameters from fits to population and cohort survival data for each genotype, with 95% C.I.'s from the Monte Carlo technique in brackets.
(PDF)

Table S2 Best-fit Dornhorst model parameters from fits to population and cohort survival data for each genotype, with 95% C.I.'s from the Monte Carlo technique in brackets.
(PDF)

Table S3 Best-fit DLS model parameters from fits to population and cohort survival data for each genotype, with 95% C.I.'s from the Monte Carlo technique in brackets.
(PDF)
