# Supplementary material for: A Model for Studying the Hemostatic Consumption or Destruction of Platelets
Source: PLoS One. 2013 Mar 7;8(3):e57783. doi: 10.1371/journal.pone.0057783 (PMC3591423; doi:10.1371/journal.pone.0057783)
Supplement: Table S1 — Best-fit LS model parameters from fits to population and cohort survival data for each genotype, with 95% C.I.'s from the Monte Carlo technique in brackets. (PDF) [file pone.0057783.s004.pdf]

Table S1. Best-fit LS model parameters from fits to population and cohort survival data for each genotype, with 95% C.I.'s from the Monte Carlo technique in brackets.

genotype	Platelet count, N (x10^3 μL^{-1})	Production rate, S (x10^3 μL^{-1}hr^{-1})	mean life span, μ (hr)	std. dev. life span, σ (hr)	mean log life span, m	std. dev. log life span, s	labelling efficiency, e_1	labelling efficiency, e_2	biotin half-life, $b_{1/2}$ (hr)
Bcl-x$^{+/Plt20}$	847 ±35	16.0 [15.6,16.5]	52.9 [51.4,54.3]	11.8 [8.1,14.7]	3.94 [3.90,3.98]	0.220 [0.151,0.277]	0.911 [0.899,0.925]	0.607 [0.573,0.643]	0.9 [0.0,2.1]
wild type	1183 ±70	14.8 [12.8,14.0]	88.8 [84.2,92.6]	28.7 [22.6,35.3]	4.44 [4.34,4.50]	0.315 [0.242,0.405]	0.879 [0.868,0.888]	0.611 [0.577,0.638]	4.0 [2.7,5.8]
Bak$^{-/-}$	1798 ±148	10.5 [10.1,11.0]	172.0 [163.1,178.4]	44.3 [35.2,57.8]	5.12 [5.04,5.16]	0.253 [0.196,0.341]	0.816 [0.806,0.826]	0.580 [0.544,0.608]	4.5 [2.9,7.5]
