# Supplementary material for: A Model for Studying the Hemostatic Consumption or Destruction of Platelets
Source: PLoS One. 2013 Mar 7;8(3):e57783. doi: 10.1371/journal.pone.0057783 (PMC3591423; doi:10.1371/journal.pone.0057783)
Supplement: Table S2 — Best-fit Dornhorst model parameters from fits to population and cohort survival data for each genotype, with 95% C.I.'s from the Monte Carlo technique in brackets. (PDF) [file pone.0057783.s005.pdf]

**Table S2.** Best-fit Dornhorst model parameters from fits to population and cohort survival data for each genotype, with 95% C.I.'s from the Monte Carlo technique in brackets.

| genotype | Platelet count, $N$ ($x10^3$ $\mu L^{-1}$) | Production rate, $S$ ($x10^3$ $\mu L^{-1}hr^{-1}$) | Natural life span, $T$ (hr) | random consumption rate constant, $r$ ($\mu L^{-1}hr^{-1}$) | random loss rate, $R$ ($\mu L^{-1}day^{-1}$) | random consumption fraction, $f$ | labelling efficiency, $e_1$ | labelling efficiency, $e_2$ | biotin half-life, $b_{1/2}$ (hr) |
|---|---|---|---|---|---|---|---|---|---|
| *Bcl-x*$^{+/Plt20}$ | 847 ±35 | 16.4 [15.5,18.2] | 55.0 [53.6,57.5] | 0.0022 [0.0000,0.0073] | 45 [0,149] | 0.12 [0.00,0.34] | 0.912 [0.900,0.928] | 0.612 [0.576,0.644] | 0.7 [0.0,2.0] |
| wild type | 1183 ±70 | 16.2 [14.9,16.4] | 111.9 [102.3,112.4] | 0.0082 [0.0053,0.0085] | 233 [149,242] | 0.60 [0.42,0.61] | 0.891 [0.880,0.896] | 0.595 [0.575,0.621] | 5.2 [3.7,6.1] |
| *Bak*$^{-/-}$ | 1798 ±148 | 12.6 [11.9,13.3] | 202.3 [192.3,209.6] | 0.0037 [0.0028,0.0045] | 159 [118,195] | 0.53 [0.42,0.61] | 0.830 [0.820,0.840] | 0.569 [0.543,0.603] | 5.7 [3.6,8.1] |
