# Supplementary material for: A Model for Studying the Hemostatic Consumption or Destruction of Platelets
Source: PLoS One. 2013 Mar 7;8(3):e57783. doi: 10.1371/journal.pone.0057783 (PMC3591423; doi:10.1371/journal.pone.0057783)
Supplement: Table S3 — Best-fit DLS model parameters from fits to population and cohort survival data for each genotype, with 95% C.I.'s from the Monte Carlo technique in brackets. (PDF) [file pone.0057783.s006.pdf]

Table S3. Best-fit DLS model parameters from fits to population and cohort survival data for each genotype, with 95% C.I.'s from the Monte Carlo technique in brackets.

genotype	Platelet count, N (x10^3 μL^{-1})	Production rate, S (x10^3 μ$L^{-1}hr^{-1}$)	mean life span, μ (hr)	std. dev. life span, σ (hr)	mean log life span, m	std. dev. log life span, s	random consumption rate constant, r (μ$L^{-1}hr^{-1}$)	random loss rate, R (μ$L^{-1}day^{-1}$)	random consumption fraction, f	labelling efficiency, e_1	labelling efficiency, e_2	biotin half-life, $b_{1/2}$ (hr)
Bcl-x$^{+/Plt20}$	847 ±35	16.0 [15.6,17.6]	52.8 [51.2,56.2]	11.3 [3.1,14.0]	3.94 [3.90,4.02]	0.211 [0.056,0.265]	0.0000 [0.0000,0.0052]	0 [0,104]	0.00 [0.00,0.25]	0.911 [0.900,0.927]	0.608 [0.573,0.643]	0.9 [0.0,2.2]
wild type	1183 ±70	14.8 [14.0,15.4]	102.3 [97.3,106.3]	23.7 [20.0,27.7]	4.60 [4.54,4.65]	0.228 [0.190,0.276]	0.0048 [0.0030,0.0063]	135 [85,178]	0.38 [0.25,0.48]	0.884 [0.876,0.892]	0.607 [0.584,0.629]	4.5 [3.4,5.7]
Bak$^{-/-}$	1798 ±148	11.9 [11.2,12.6]	192.3 [182.3,201.1]	35.6 [23.8,44.9]	5.24 [5.18,5.29]	0.183 [0.120,0.242]	0.0026 [0.0014,0.0035]	110 [59,150]	0.39 [0.22,0.50]	0.826 [0.816,0.836]	0.570 [0.544,0.607]	5.7 [3.4,7.7]
